# Post-weaning diarrhea caused by F18^+^ *Escherichia coli* and its impact on mucosa-associated microbiota and immune responses in the jejunum of nursery pigs

**DOI:** 10.20517/mrr.2025.14

**Published:** 2025-07-30

**Authors:** Jeonghyeon Son, Sung Woo Kim

**Affiliations:** Department of Animal Science, North Carolina State University, Raleigh, NC 27695, USA.

**Keywords:** F18^+ ^*Escherichia coli*, intestinal health, jejunum, nursery pigs, post-weaning diarrhea

## Abstract

This review examines the impacts of F18^+^ *Escherichia coli* (*E. coli*) on the mucosa-associated microbiota, mucosal immune responses, oxidative stress, and intestinal morphology in the jejunum of nursery pigs. F18^+^ *E. coli* is a major cause of post-weaning diarrhea (PWD) in nursery pigs, mainly due to the production of enterotoxins that disrupt electrolyte balance in the intestinal lumen, leading to diarrhea, growth retardation, increased mortality, and economic losses. Experimental F18^+^ *E. coli* challenge models have shown an increased incidence of diarrhea (28.3%), along with reductions in average daily gain (24.1%), average daily feed intake (12.5%), and gain-to-feed ratio (14.9%). These adverse effects are largely attributed to microbial dysbiosis and heightened mucosal immune responses in the jejunum. The F18^+^ *E. coli* challenge also increases the abundance of harmful bacteria while reducing beneficial bacteria in the jejunal mucosa. Research using this challenge model has demonstrated elevated levels of tumor necrosis factor-α (14.9%), interleukin-8 (10.9%), immunoglobulin A (9.2%), immunoglobulin G (19.7%), malondialdehyde (50.7%), and protein carbonyls (32.3%). These immune and oxidative responses are associated with reductions in villus height (VH) (10.2%) and VH-to-crypt depth ratio (10.7%), as well as an increase in Ki-67^+^ proliferative cells (35.4%) in the jejunum. In conclusion, F18^+^ *E. coli* induces PWD and compromises intestinal health in nursery pigs through dysbiosis, inflammation, oxidative stress, and morphological changes, ultimately impairing growth.

## INTRODUCTION

Maintaining intestinal health is important for the growth of pigs, as the intestine is the primary site for nutrient digestion and absorption^[1,2]^. It also plays a key role in immune function, housing a large number of immune cells in pigs^[3-5]^. However, the pig intestine is highly susceptible to infection during certain stages, especially the nursery period, when it is still immature. During early weaning, piglets are unable to secrete sufficient digestive enzymes, resulting in incomplete nutrient hydrolysis and the accumulation of undigested nutrients in the intestine. This accumulation alters the intestinal microbiota by promoting the colonization of pathogenic bacteria, which triggers intestinal inflammation^[6,7]^. The resulting inflammatory response increases oxidative stress, leading to damage of the intestinal tissue^[8,9]^. Consequently, nutrient absorption is impaired, delaying pig growth. During this vulnerable period, additional diseases can further compromise intestinal health.

One major pathogen that affects intestinal health during this stage is enterotoxigenic *Escherichia coli* (*E. coli*), to which nursery pigs are particularly susceptible. This infection significantly impairs growth and productivity. Unlike other species, pigs express F18 receptors on the epithelial cells of the small intestine, allowing the F18 fimbriae of enterotoxigenic *E. coli* to adhere^[10,11]^. Enterotoxigenic *E. coli* is classified into two major types - F18^+^ and F4^+^ *E. coli* - based on their fimbrial antigens^[12]^. A recent study reported an increasing trend in F18*^+^* *E. coli* infections, while F4^+^ infections have remained relatively stable^[13]^. This shift may be attributed to modern commercial weaning practices and differences in antimicrobial susceptibility. Notably, the genes encoding F18 receptors are highly expressed between 3 and 4 weeks of age^[14]^, which coincides with the typical weaning period in commercial pig production. This may explain the heightened susceptibility of piglets to F18^+^ *E. coli* infection during weaning^[14]^. The F18 fimbriae of F18^+^ *E. coli* facilitate attachment to the epithelial F18 receptors, promoting colonization and the secretion of enterotoxins^[15]^. While the antimicrobial susceptibility of F4^+^ *E. coli* has remained stable, resistance has increased among F18^+^ strains, potentially contributing to their rising prevalence^[13]^. The enterotoxins secreted by F18^+^ *E. coli* disrupt electrolyte balance, causing water efflux from epithelial cells into the intestinal lumen, which leads to post-weaning diarrhea (PWD). For these reasons, F18^+^ *E. coli* is now considered the primary cause of PWD among enterotoxigenic strains^[11]^. In pigs challenged with F18^+^ *E. coli*, the incidence of diarrhea increased by 28.3%, and fecal scores rose by 0.7 on a 1-to-5 scale (1: very firm stool; 5: very watery stool), compared to unchallenged pigs [Table 1]^[16-20]^.

**Table 1:** Changes in diarrhea incidence and fecal score in nursery pigs challenged with F4^+^ or F18^+^ *E. coli*

**Ref.**	**IBW (kg)**	**Experimental period (d)**	**Change**	
			**Incidence of diarrhea (%)^1^**	**Fecal score^2^**
F4^+^ *E. coli*				
Molist *et al.*^[21]^	5.0	16	-	–0.800^*^
Yi *et al.*^[16]^	5.3	14	-	0.644
Silveira *et al.*^[22]^	6.4	21	9.4^*^	-
Khafipour *et al.*^[17]^	6.8	20	-	1.127^*^
F18^+^ *E. coli*				
Duarte *et al.*^[23]^	5.8	28	-	0.913
Heo *et al.*^[24]^	5.9	28	20.0^*^	-
He *et al.*^[25]^	6.2	28	-	0.524
Liu *et al.*^[26]^	6.3	15		0.885^*^
Duarte *et al.*^[9]^	6.3	28	-	1.336
Becker *et al.*^[18]^	6.4	17	-	1.239
Garavito-Duarte *et al.*^[27]^	6.4	28	-	0.200
Gormley *et al.*^[28]^	6.4	28	49.9^*^	-
Jang *et al.*^[29]^	6.5	28	-	0.300^*^
Xu *et al.*^[30]^	6.6	28	-	0.320
Deng *et al.*^[31]^	6.6	25	-	0.133
Kim *et al.*^[32]^	6.7	18	-	0.910
Li *et al.*^[33]^	6.9	17	32.9^*^	-
Sun *et al.*^[19]^	7.0	21	-	0.980^*^
Wong *et al.*^[34]^	7.2	28	10.4^*^	-
Jinno *et al.*^[35]^	7.4	28	-	0.405
Duarte *et al.*^[36]^	7.9	20	-	0.985
Duarte *et al.*^[37]^	7.9	28	-	0.170
Chang *et al.*^[20]^	8.0	16	-	1.067^*^
**Causes**	**Number of studies^3^**		**Average change^4^**	
	**Incidence of diarrhea**	**Fecal score**	**Incidence of diarrhea (%)**	**Fecal score**
F4^+^ and F18^+^ *E. coli*	5	18	24.5	0.630
F4^+^ *E. coli*	1	3	9.4	0.323
F18^+^ *E. coli*	4	15	28.3	0.691

1 Diarrhea incidence (%) was calculated as the difference between the positive control (*E. coli*-challenged group) and the negative control (non-challenged group). ^2^Fecal score changes represent the difference in average fecal scores between the positive and negative control groups, standardized to a 1 to 5 scale. Scores originally reported on a 0 to 3 or 1 to 4 scale were converted to a 1 to 5 scale. ^3^The number of studies reflects those included in calculating the average change in diarrhea incidence and fecal score. ^4^Average changes were calculated as arithmetic means. ^*^P < 0.05. *E. coli*: *Escherichia coli*; IBW: initial body weight.

In addition, F18^+^ *E. coli*-induced PWD significantly impaired growth performance in nursery pigs, with reductions in average daily gain, average daily feed intake (ADFI), and gain-to-feed ratio (G:F) by 24.1%, 12.5%, and 14.9%, respectively [Table 2]. Moreover, pigs suffering from PWD due to F18^+^ *E. coli* exhibit impaired intestinal morphology and a leaky gut, further heightening their susceptibility to secondary infections and resulting in increased veterinary costs^[38]^.

**Table 2:** Changes in growth performance in nursery pigs challenged with F4^+^ or F18^+^ *E. coli*

**Ref.**	**IBW (kg)**	**Experimental period (d)**	**Change^1^ (Δ%)**		
			**ADG**	**ADFI**	**G:F**
F4^+^ *E. coli*					
Yi *et al.*^[16]^	5.3	14	–51.4	–6.6	–48.0^*^
Silveira *et al.*^[22]^	6.4	21	–1.8	0.3	–2.1
Khafipour *et al.*^[17]^	6.8	20	–0.6	–0.7	0.1
F18^+^ *E. coli*					
Heo *et al.*^[24]^	5.9	28	–31.2^*^	–9.9^*^	–23.6^*^
He *et al.*^[25]^	6.2	28	–28.6^*^	–11.4	–19.7^*^
Liu *et al.*^[26]^	6.3	15	–28.3^*^	10.6	–35.2^*^
Duarte *et al.*^[9]^	6.3	28	–21.8^*^	–9.5	–22.2^*^
Becker *et al.*^[18]^	6.4	17	–51.3^*^	–32.7^*^	–33.3
Garavito-Duarte *et al.*^[27]^	6.4	28	–24.0^*^	–26.2^*^	–14.9^*^
Gormley *et al.*^[28]^	6.4	28	–19.6^*^	–21.6^*^	1.6
Jang *et al.*^[29]^	6.5	28	–0.8	5.5	–0.61
Xu *et al.*^[30]^	6.6	28	–23.9	–18.1	–14.1
Deng *et al.*^[31]^	6.6	25	–32.0^*^	–28.8^*^	–5.7
Kim *et al.*^[32]^	6.7	18	–34.6	–17.8	–23.5
Li *et al.*^[33]^	6.9	17	–41.4^*^	–31.1^*^	–20.3
Sun *et al.*^[19]^	7.0	21	–8.3	–2.5	–6.7
Wong *et al.*^[34]^	7.2	28	–19.5^*^	–11.2	–9.7
Jinno *et al.*^[35]^	7.4	28	–16.9^*^	–9.9	–9.4
Duarte *et al.*^[36]^	7.9	20	–16.8^*^	–5.6	–13.0^*^
Duarte *et al.*^[37]^	7.9	28	–2.9	–0.2	–1.4
Chang *et al.*^[20]^	8.0	16	–31.0^*^	–5.0^*^	–27.4^*^
**Causes**		**Number of studies^2^**	**Average change^3^ (Δ%)**		
			**ADG**	**ADFI**	**G:F**
F4^+^ and F18^+^ *E. coli*		21	–23.2	–11.1	–15.2
F4^+^ *E. coli*		3	–17.9	–2.3	–16.7
F18^+^ *E. coli*		18	–24.1	–12.5	–14.9

Percentage changes in growth performance metrics were calculated by comparing values from the positive control (*E. coli*-challenged group) and the negative control (non-challenged group). ^2^The number of studies included refers to those used to determine the average change in growth parameters. ^3^Average changes were calculated as arithmetic means. ^*^P < 0.05. *E. coli*: *Escherichia coli*; IBW: initial body weight; ADG: average daily gain; ADFI: average daily feed intake; G:F: gain-to-feed ratio.

F18^+^ *E. coli* infection can further compromise intestinal health in pigs already stressed by weaning. As a result, the F18^+^ *E. coli* challenge model has been widely used in nursery pigs to evaluate its effects on intestinal health^[23-26]^. Traditionally, fecal or digesta microbiota have been assessed as indicators of intestinal health in pigs^[39,40]^. However, recent research suggests that mucosa-associated microbiota - directly collected from the mucosa layer - provide a more reliable indicator^[6,41]^. This is because mucosa-associated microbiota are more intimately linked with epithelial cells than luminal microbiota, offering greater potential for host-microbe interactions^[8,42,43]^.

The jejunum has been highlighted as a key site for investigating microbial dynamics and epithelial immune responses. Studies have utilized jejunal mucosa and tissues to evaluate intestinal health in nursery pigs^[2,12,23,44,45]^. As the longest segment of the gastrointestinal tract, the jejunum is the first to encounter external substances from feed, including not only nutrients but also anti-nutritional and allergenic compounds that can trigger immune defenses before these reach the hindgut^[31,44]^. Consequently, the jejunum is more vulnerable to these external factors than the large intestine. It also plays a central role in digestion and nutrient absorption and contains immune cells in quantities comparable to other intestinal regions^[46-48]^. Moreover, the small intestine harbors a lower microbial density than the large intestine, making it more susceptible to the direct effects of F18^+^ *E. coli*. Given these characteristics, the jejunum is a critical focus for investigating intestinal health. This review aims to explore the mechanisms and effects of F18^+^ *E. coli* infection on jejunal mucosa and tissue in nursery pigs, with particular attention to mucosa-associated microbiota, mucosal immune responses, oxidative stress, and morphological changes. While numerous studies have reviewed dietary strategies to mitigate F18^+^ *E. coli* infection^[12,45,49]^, this review emphasizes the underlying mechanisms and pathological impacts of the infection as it relates to PWD.

## RECOGNITION AND INTERACTION OF F18^+^ *E. coli* WITH JEJUNAL EPITHELIAL CELLS

**Figure 1:**
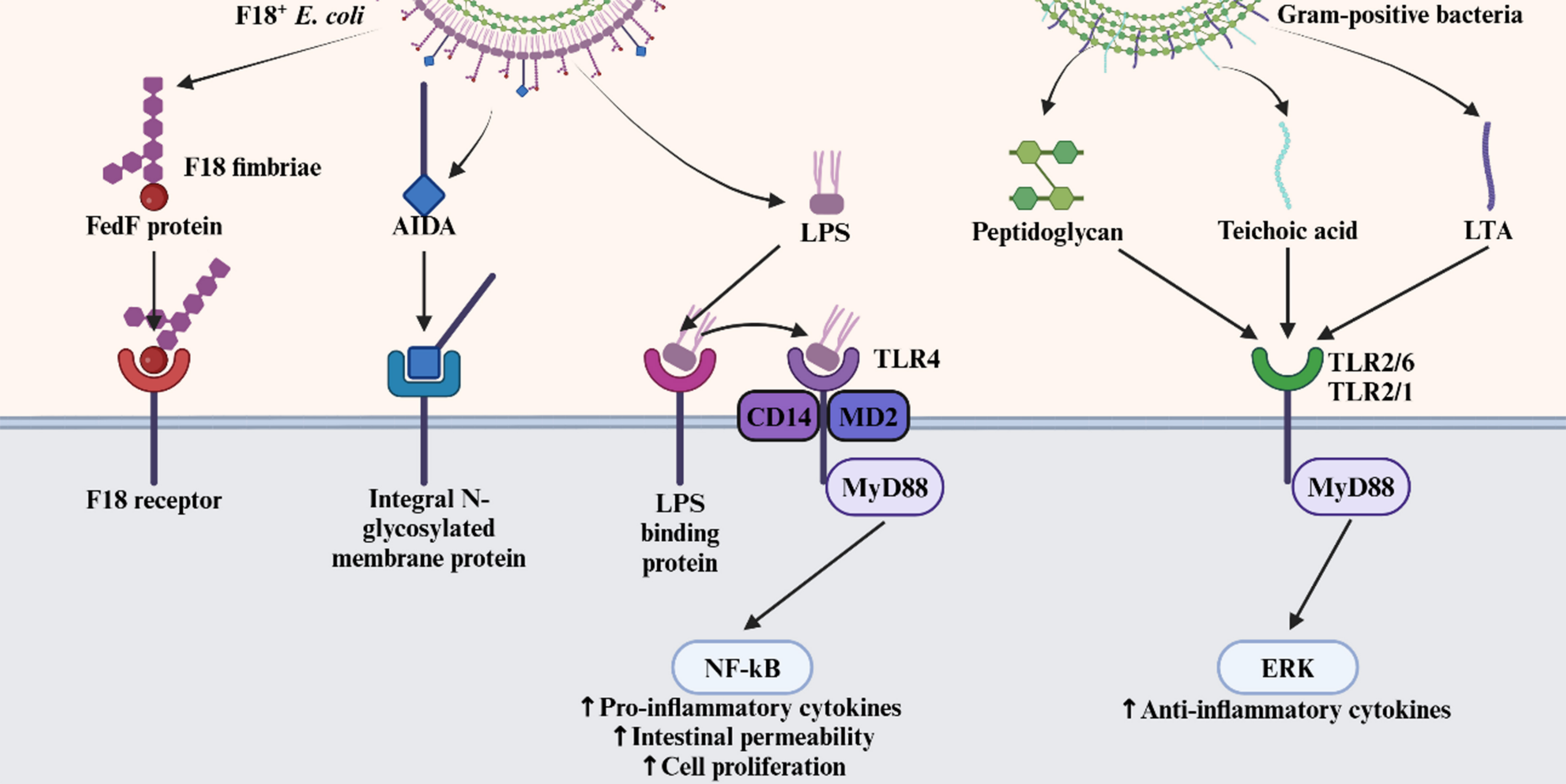
Recognition and interaction of F18^+^ *E. coli* and Gram-positive bacteria with epithelial cells through PRRs. The F18 fimbriae, unique to F18^+^ *E. coli*, bind to the F18 receptor via the FedF protein. Additionally, the diffusely adhering adhesin binds to an integral N-glycosylated membrane protein. These adhesins enhance bacterial adhesion and promote colonization. LPS, a component of the F18^+^ *E. coli* outer membrane, is recognized by PRRs. Initially, LPS binds to LPS-binding protein and is then transferred to CD14, which delivers it to the TLR4/MD2 complex. This interaction activates the MyD88-dependent pathway, leading to the release of NF-κB. Activated NF-κB promotes the secretion of pro-inflammatory cytokines and increases intestinal permeability, leading to diarrhea in pigs and enhanced epithelial cell proliferation. In contrast, peptidoglycan from Gram-positive bacteria is recognized by TLR2. Teichoic acid and LTA, unique to Gram-positive bacteria, are also recognized by TLR2. These recognitions activate the MyD88-dependent pathway and stimulate the ERK pathway, resulting in the secretion of anti-inflammatory cytokines and reduced inflammation. Created in BioRender. Son, J. (2025) https://BioRender.com/950e0xm. *E. coli*: *Escherichia coli*; PRRs: pattern recognition receptors; LPS: lipopolysaccharide; CD14: cluster of differentiation 14; TLR4: Toll-like receptor 4; MD2: myeloid differentiation factor 2; MyD88: myeloid differentiation primary response 88; NF-κB: nuclear factor kappa B; TLR2: Toll-like receptor 2; LTA: lipoteichoic acid; ERK: extracellular signal-regulated kinase.

The recognition of F18^+^ *E. coli* is the initial step in its interaction with epithelial cells and is critical for adhesion and inflammation. This process occurs in the small intestine of pigs^[50]^. Bacterial recognition by epithelial cells depends on structural components of the bacteria [Figure 1]. F18^+^ *E. coli* is a Gram-negative bacterium distinguished by the presence of F18 fimbriae and lipopolysaccharide (LPS) in its outer membrane. The F18 fimbriae, uniquely expressed by F18^+^ *E. coli*, contain Fed proteins, among which FedF mediates binding to specific epithelial cell receptors^[12,51,52]^. Once bound, the positively charged lysine residues in the F18 receptor interact with the bacterial membrane, stabilizing the attachment between the receptor and F18 fimbriae^[15]^. In addition, an adhesin involved in diffuse adherence - a different adhesin protein found in *E. coli* - binds to an integral N-glycosylated membrane protein, further facilitating bacterial adhesion to host epithelial cells^[53]^. These adhesins enable F18^+^ *E. coli* to tightly attach to the intestinal epithelium, proliferate, and colonize the small intestine.

LPS functions as a pathogen-associated molecular pattern recognized by pattern recognition receptors (PRRs) such as Toll-like receptor 4 (TLR4), initiating inflammation in the pig intestine. LPS recognition begins with its capture by LPS-binding protein, which transfers it to the cluster of differentiation 14 (CD14)^[54]^. CD14 subsequently delivers LPS to the TLR4 and myeloid differentiation factor 2 (MD2) complex, triggering receptor activation^[54]^. This activates the myeloid differentiation primary response 88 (MyD88)-dependent signaling pathway, which in turn stimulates the nuclear factor kappa B (NF-κB) pathway^[55]^. Activation of NF-κB leads to the production of pro-inflammatory cytokines, including tumor necrosis factor-alpha (TNF-α), interleukin-6 (IL-6), and interleukin-8 (IL-8) in the epithelial cytoplasm^[55]^. These pro-inflammatory cytokines, induced by Gram-negative bacteria, downregulate ion transporter gene expression in intestinal cells, impairing electrolyte balance in the lumen, and contribute to diarrhea in pigs^[56]^. Additionally, NF-κB–mediated inflammation damages tight junction proteins between epithelial cells, increasing intestinal permeability and facilitating water loss^[57-59]^. Activated NF-κB also promotes epithelial cell proliferation for tissue repair by influencing the mechanistic target of rapamycin (mTOR) pathway. Specifically, NF-κB suppresses tuberous sclerosis complex 2, a negative regulator of mTOR complex 1^[60]^, thereby enhancing mTOR signaling and driving cell proliferation^[60]^.

Peptidoglycan, another bacterial cell wall component, is found in both Gram-negative and Gram-positive bacteria. However, in F18^+^ *E. coli*, peptidoglycan is shielded by LPS and is therefore less effectively recognized by PRRs in epithelial cells than in Gram-positive bacteria. The cell wall of Gram-positive bacteria also contains teichoic acid and lipoteichoic acid, which are recognized by Toll-like receptor 2 (TLR2), either as TLR2/TLR1 or TLR2/TLR6 heterodimer located on the apical surface of epithelial cells^[61,62]^. Thus, TLR2-mediated recognition is primarily associated with Gram-positive bacteria. This recognition activates the MyD88-dependent signaling cascade and the extracellular signal-regulated kinase (ERK) pathway, promoting the secretion of anti-inflammatory cytokines such as interleukin-10, which help reduce intestinal inflammation.

## MECHANISMS OF F18^+^ *E. coli*-INDUCED PWD IN PIGS

**Figure 2:**
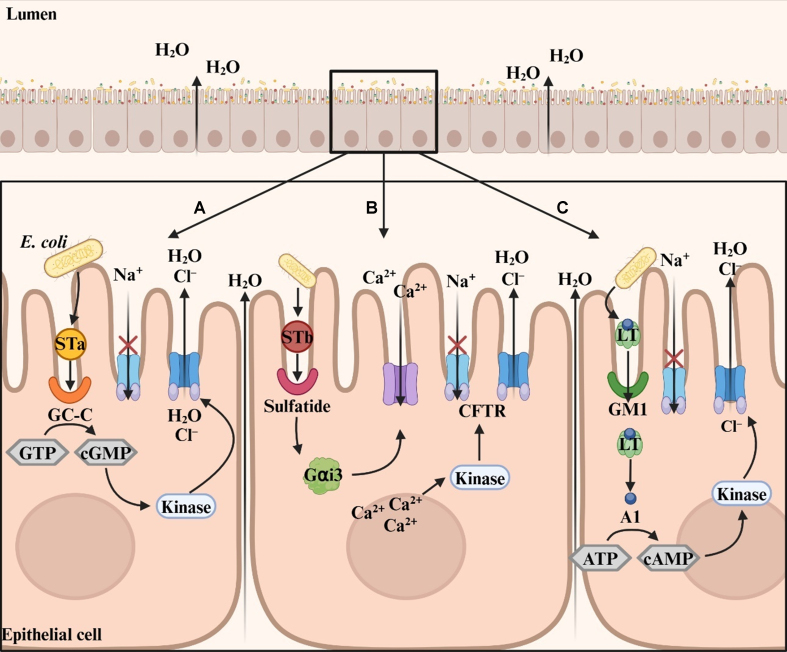
Mechanism of PWD induced by three toxins secreted by enterotoxigenic *E. coli*. (A) STa binds to the GC-C receptor, activating its catalytic domain and converting GTP into cGMP. Elevated cGMP levels activate kinases that phosphorylate the regulatory domain of CFTR, enhancing Cl^-^ secretion and inhibiting Na^+^ absorption, thereby disrupting electrolyte balance; (B) STb binds to sulfatide receptors, activating the GTP-binding protein Gαi3, which increases intracellular Ca^2+^ via Ca^2+^ channels. The elevated Ca^2+^ levels activate kinases that affect electrolyte transport; (C) LT binds to the GM1 receptor on epithelial cells, facilitating its entry. Once internalized, the A1 subunit of LT converts ATP into cAMP. Increased cAMP activates kinases that phosphorylate CFTR, exacerbating electrolyte imbalance. The cumulative effects of these toxins disrupt ion transport, promoting water secretion into the small intestine and resulting in diarrhea. Created in BioRender. Son, J. (2025) https://BioRender.com/j39i382. PWD: Post-weaning diarrhea; *E. coli*: *Escherichia coli*; STa: heat-stable toxin a; GC-C: guanylate cyclase C; GTP: guanosine triphosphate; cGMP: cyclic guanosine monophosphate; CFTR: cystic fibrosis transmembrane conductance regulator; STb: heat-stable toxin b; LT: heat-labile toxin; GM1: monosialotetrahexosylganglioside; ATP: adenosine triphosphate; cAMP: cyclic adenosine monophosphate.

F18^+^ *E. coli* secretes several toxins in the small intestine, including heat-labile toxin (LT), heat-stable toxin (ST), Shiga toxin, and enteroaggregative *E. coli* heat-stable enterotoxin^[11]^. Among these, the major toxins responsible for PWD are ST and LT, both of which disrupt electrolyte balance, ultimately leading to diarrhea. ST toxins exist in two forms: heat-stable toxin a (STa) and heat-stable toxin b (STb). The receptor for STa is located on the brush border membrane of enterocytes and is known as transmembrane guanylate cyclase C [Figure 2A]^[63]^. Upon STa binding, the intracellular catalytic domain of the receptor is activated, converting guanosine triphosphate (GTP) into cyclic guanosine monophosphate (cGMP). Increased cGMP levels activate both cyclic adenosine monophosphate (cAMP)-dependent protein kinase A and cGMP-dependent protein kinase II^[64]^. These kinases phosphorylate the regulatory domain of cystic fibrosis transmembrane conductance regulator (CFTR), altering its conformation and ion channel function. This results in increased Cl^-^ secretion and decreased Na^+^ absorption^[65]^. The resulting electrolyte imbalance creates an osmotic gradient that drives water into the intestinal lumen, leading to watery diarrhea in pigs. In contrast, the receptor for STb is sulfatide, an acidic glycosphingolipid located on the apical membrane of enterocytes [Figure 2B]^[66,67]^. STb binding activates a GTP-binding regulatory protein, which increases intracellular Ca^2+^ levels through ligand-gated Ca^2+^ channels^[66]^. Elevated Ca^2+^ activates calmodulin-dependent protein kinase II, which in turn phosphorylates CFTR and modulates electrolyte channels^[68]^. This signaling cascade enhances Cl^-^ efflux and promotes water movement into the intestinal lumen, contributing to PWD.

LT contributes to electrolyte imbalance and water secretion in a mechanism similar to that of ST, primarily through CFTR activation in the small intestine. Structurally, LT consists of an A subunit and a pentameric B subunit^[69]^. The B subunit binds to the monosialotetrahexosylganglioside (GM1) receptor on the mucosal surface of the small intestine^[70]^, facilitating endocytosis of LT into epithelial cells [Figure 2C]. Inside the cell, the A subunit is cleaved into A1 and A2 fragments^[70]^. The A1 fragment increases intracellular cAMP levels in the cytoplasm^[71]^, which then activates protein kinase A. This kinase phosphorylates CFTR, leading to altered electrolyte transport and ultimately causing PWD in nursery pigs.

## F18^+^ *E. coli* CHALLENGE MODEL AND ITS IMPORTANCE

F18^+^ *E. coli* infection is commonly observed during the nursery phase on commercial pig farms. To mitigate its negative impacts, researchers have developed dietary interventions targeting this pathogen^[30,34,35,72,73]^. The efficacy of these interventions is typically evaluated using the F18^+^ *E. coli* challenge model, which aims to replicate conditions found in commercial settings. Several factors must be considered to accurately mimic F18^+^ *E. coli* infection under such conditions. Pigs used in the challenge model should express the F18 receptor, which facilitates the attachment and colonization of F18^+^ *E. coli*. Previous studies have identified the FUT1 gene as being associated with the expression of this receptor, indicating that genetic variations in *FUT1* may influence pigs’ susceptibility to infection^[51,74]^. Commercial pig breeds are commonly used in these studies. Although genetically modified pigs lacking the F18 receptor have been developed to improve resistance to F18^+^ *E. coli*^[75,76]^, they may not be representative of commercial pigs, which are primarily selected for growth performance and meat quality.

The timing of inoculation is another critical factor influencing susceptibility to infection. When pigs are weaned at 3 weeks of age, inoculation 1 week after weaning is recommended. Expression of the F18 receptor begins around 10 days of age and gradually increases with age^[14]^. However, the age at which expression reaches a plateau has varied across studies^[10,14]^. Some studies have opted to inoculate pigs 3-5 days post-weaning to capitalize on the stress experienced immediately after weaning^[20,24,26]^. However, this approach may reduce the effectiveness of the challenge due to the lingering passive immunity provided by sow’s milk. Conversely, inoculating too late may reduce susceptibility due to the pigs’ adaptation to post-weaning stress. Therefore, inoculation at 7 days post-weaning (based on an average age of 21 days at weaning) is considered optimal in the F18^+^ *E. coli* challenge model.

Inoculation method and dosage are also key variables affecting the success of F18^+^ *E. coli* infection. To improve consistency, it is recommended to divide the inoculation into multiple doses while monitoring pig responses, rather than administering a single high dose. This approach accounts for individual variation in susceptibility. A single high dose may not reliably induce infection across all pigs. To mitigate this issue, 3-4 separate inoculations are often employed. Previous studies have used between 1 and 4 inoculations to ensure successful infection^[9,27-29]^. If F18^+^ *E. coli* colonization is successful, clinical signs such as diarrhea may appear within 24 h^[18,36]^. Additional symptoms include feed refusal, lethargy, sunken eyes, droopy ears, and elevated body temperature, often occurring before the onset of diarrhea. These indicators can be used to assess infection. Based on observed responses, additional inoculations may be necessary to ensure consistent infection and to avoid false-negative results in intervention studies. Regarding dosage, no universally accepted standard exists for inducing diarrhea. Reported dosages to induce PWD have ranged from 1.9 × 10^8^ to 3 × 10^10^ CFU^[24,25,34,35]^. Thus, administering multiple moderate doses rather than a single high dose is recommended to reduce variability in infection outcomes.

## THE IMPORTANCE OF RESEARCH ON JEJUNAL MUCOSA-ASSOCIATED MICROBIOTA AND IMMUNITY IN PIGS

Intestinal health is crucial for pig production, as it directly influences nutrient utilization and growth performance. When intestinal health is compromised, nutrients that would otherwise support growth are redirected toward tissue repair and immune responses, ultimately reducing growth efficiency and productivity. Historically, antimicrobial growth promoters (AGPs) were widely used to maintain intestinal health in pigs^[77]^. However, growing concerns over antimicrobial resistance have led to a decline in AGP usage, exacerbating intestinal health challenges. Consequently, increasing research attention has focused on maintaining intestinal health without the use of AGPs^[2,39,40]^.

Both the composition of the microbiota and intestinal immunity are critical determinants of intestinal health and must be understood to improve pig health and performance. The intestinal microbiota not only affects nutrient digestion but also modulates immune responses. Moreover, the intestine is the largest immune organ in pigs and plays a key role in protecting against pathogens. Considering the essential roles of both microbiota and immunity, selecting the appropriate intestinal region for research is crucial. Among the various intestinal segments, the jejunum is particularly important for investigating intestinal immunity in pigs. It is the first site where the immune system interacts with ingested materials, including pathogens. Additionally, the jejunum contains both isolated and distributed Peyer’s patches, indicating consistent immune activity along its length^[3]^. Several immune-related genes, including C-C chemokine receptor type 10 and IL-15, are more highly expressed in the jejunum than in the ileum in 28-day-old pigs^[78]^. As the longest portion of the small intestine, the jejunum is also the primary location for nutrient digestion and absorption in pigs^[2,79]^. It has more villi and microvilli than the large intestine, resulting in a greater surface area. Most nutrients are absorbed in the jejunum, with only the remaining portion passed to the ileum and large intestine. Additionally, the small intestine harbors a lower concentration of microbiota compared to the large intestine, making it more vulnerable to disturbances, such as those caused by F18^+^ *E. coli* in challenge models.

In the jejunum, mucosa-associated microbiota are more directly relevant to intestinal health than luminal microbiota, primarily due to their interaction with enterocyte PRRs. Mucosa-associated microbiota are in close contact with epithelial cells and influence the intestinal immune system, whereas luminal microbiota interact less directly with epithelial cells and primarily affect the digesta^[80]^. Furthermore, the composition of mucosa-associated microbiota differs from that of luminal microbiota. Recent studies have emphasized the distinction between these two microbial populations due to differing environmental characteristics in the lumen and mucosa, such as pH and oxygen levels^[9,23,81]^. Luminal microbiota are more sensitive to dietary changes and environmental fluctuations than mucosa-associated microbiota. For instance, the pH in the jejunal lumen is more variable due to the influence of intestinal secretions like bile acids, while the pH at the mucosal surface is relatively stable to protect epithelial tissues. Oxygen levels also differ, with higher oxygen concentrations near the mucosa due to vascularization^[82]^. As a result, luminal microbiota are mainly anaerobic, whereas mucosa-associated microbiota are more often aerobic^[83,84]^. Given these characteristics, the jejunal mucosa-associated microbiota serve as a valuable indicator for evaluating intestinal health in pigs.

## IMPACTS OF F18^+^ *E. coli* CHALLENGE ON THE JEJUNAL MUCOSA-ASSOCIATED MICROBIOTA

As mentioned previously, the intestinal microbiota interacts with intestinal cells in pigs both directly and indirectly. The composition of the jejunal mucosa-associated microbiota serves as an important indicator of intestinal health in pigs. Data from eight studies investigating the impact of F18^+^ *E. coli* challenge on the jejunal mucosa-associated microbiota were analyzed, summarizing changes in relative abundance (RA) at the phylum, family, genus, and species levels [Table 3]^[9,23,27-30,36,37]^. Literature was retrieved from PubMed and Google Scholar using keywords such as F18^+^ *E. coli*, challenge, PWD, mucosa-associated microbiota, intestinal health, jejunum, and nursery pigs. The studies were manually screened based on their experimental procedures. For consistency, only studies involving both negative control (NC) and positive control groups, and using nursery pigs, were included. Data derived from jejunal digesta were excluded. Only results showing statistically significant changes are presented.

**Table 3:** Changes in RA of jejunal mucosa-associated microbiota in nursery pigs challenged with F18^+^ *E. coli*

**Ref.**	**Inoculation BW (kg)**	**Inoculation age (d)**	**Days post inoculation (d)**	**Response^1^**			
				**Phylum**	**Family**	**Genus**	**Species**
Duarte and Kim^[23]^	NA	28	21	No effect	Helicobacteraceae ↑ Pseudomonadaceae ↑ Xanthomonadaceae ↑ Peptostreptococcaceae ↑ Campylobacteraceae ↓ Enterobacteriaceae ↓ Brachyspiraceae ↓ Caulobacteraceae ↓	*Helicobacter* ↑ *Campylobacter* ↓	*Helicobacter rappini* ↑ *Helicobacter equorum* ↑^#^ *Streptococcus hyointestinalis* ↑^#^ *Campylobacter coli* ↓ *Acinetobacter lwoffii* ↓
Garavito-Duarte *et al.*^[27]^	6.3	28	21	Bacteroidetes ↑ Spirochaetae ↑^#^	Prevotellaceae ↑ Lachnospiraceae ↑ Ruminococcaceae ↑ Lactobacillaceae ↓^#^ Bifidobacteriaceae ↓^#^	*Lactobacillus* ↓^#^ *Bifidobacterium* ↓^#^	*Helicobacter equorum* ↑ *Bifidobacterium dentium* ↓ *Lactobacillus delbrueckii* ↓ *Bifidobacterium boum* ↓ *Lactobacillus salivarius* ↓
Gormley *et al.*^[28]^	6.4	28	21	-	-	*Bradyrhizobium* ↑ *Eubacterium* ↑ *Romboutsia* ↑ *Selenomonas* ↑ *Terrisporobacter* ↑ *Prevotella* ↑ *Solobacterium* ↓ *Bacillus* ↓ *Corynebacterium* ↓	*-*
Duarte *et al.*^[9]^	6.9	28	21	Bacteroidetes ↓^#^	Acidaminococcaceae ↓^#^	-	*Prevotella stercorea* ↓ *Phascolarctobacterium succinatutens* ↓^#^ *Lactobacillus delbrueckii* ↓^#^ *Prevotella copri* ↓^#^
Jang *et al.*^[29]^	7.2	28	21	No effect	No effect	-	-
Xu *et al.*^[30]^	7.1	28	21	No effect	Campylobacteraceae ↓^#^	*Selenomonas* ↓	*Helicobacter rodentium* ↑
Duarte *et al.*^[36]^	8.2	28	13	Proteobacteria ↑ Bacteroidetes ↓ Firmicutes ↓	Helicobacteraceae ↑^#^ Prevotellaceae ↓^#^ Veillonellaceae ↓^#^ Clostridiaceae ↓^#^	*Helicobacter* ↑^#^ *Mitsuokella* ↓ *Selenomonas* ↓ *Megasphaera* ↓	*Prevotella copri* ↓^#^ *Selenomonas bovis* ↓ *Roseburia faecis* ↓ *Selenomonas lipolytica* ↓ *Acidaminococcus fermentans* ↓
Duarte *et al.*^[37]^	8.2	NA	21	Proteobacteria ↑ Bacteroidetes ↓ Firmicutes ↓	Helicobacteraceae ↑ Prevotellaceae ↓ Lactobacillaceae ↓ Lachnospiraceae ↓^#^ Campylobacteraceae ↓^#^	-	*Helicobacter mastomyrinus* ↑ *Prevotella copri* ↓ *Prevotella stercorea* ↓ *Roseburia faecis* ↓

1 All results represent statistically significant changes (*P* < 0.05) in the positive control (*E. coli-*challenged group) relative to the negative control (non-challenged group), except for those marked with a hash (#), which indicate a statistical tendency (*P* < 0.10). RA: Relative abundance; *E. coli*: *Escherichia coli*; BW: body weight; NA: not available.

The F18^+^ *E. coli* challenge negatively affects the composition of the jejunal mucosa-associated microbiota in two major ways. First, it increases the RA of harmful bacteria within the jejunal mucosa, many of which are pathogenic or promote inflammation. For instance, increased RA of *Helicobacteraceae* and *Helicobacter* was observed in studies using the F18^+^ *E. coli* challenge model^[23,36]^. These taxa are associated with subclinical inflammation^[85]^. The inoculation also led to increased RA of *Pseudomonadaceae*^[23]^, a family that includes *Pseudomonas aeruginosa*, an opportunistic pathogen detrimental to intestinal health^[86]^. Additionally, an increase in *Eubacterium* RA was reported^[28]^, which may raise disease susceptibility, as certain species of *Eubacterium* are considered disease markers and potential opportunistic pathogens^[87,88]^. An elevated RA of *Terrisporobacter* was also noted, and this genus has been correlated with C-reactive protein, a well-known marker of inflammation^[89]^.

Second, F18^+^ *E. coli* reduces the RA of beneficial bacteria, contributing to dysbiosis. In one study, pigs challenged with F18^+^ *E. coli* showed a decreased RA of *Lactobacillus* and *Bifidobacterium*, which are known to produce lactic acid and short-chain fatty acids (SCFA)^[27]^. Gormley *et al.* reported that RA of *Bacillus* and *Corynebacterium*, both of which help reduce oxidative stress and produce SCFA, was lower in challenged pigs compared to NC pigs^[28]^. Similarly, F18^+^ *E. coli* appeared to reduce the RA of *Mitsuokella*, a genus capable of producing SCFA from phytate degradation^[36,90]^. RA of *Megasphaera*, which converts lactic acid into SCFAs and competes with lactic-acid-consuming harmful bacteria, was also reduced^[91,92]^. A decrease in Prevotellaceae RA was reported^[36]^, and at the species level, RA of *Prevotella copri*, a fiber-fermenting and SCFA-producing species, was found to be lower^[36]^. These reductions in SCFA-producing bacteria may lead to decreased SCFA levels and increased jejunal pH, creating favorable conditions for pathogenic bacteria. Moreover, reduced SCFA availability - essential for enterocytes - can heighten susceptibility to inflammation and disease. With respect to diarrhea, Gormley *et al.* reported that F18^+^ *E. coli* challenge increased the incidence of diarrhea alongside changes in the jejunal mucosa-associated microbiota^[28]^. Specifically, RA of *Bradyrhizobium*, *Eubacterium*, *Romboutsia*, *Selenomonas*, *Terrisporobacter*, and *Prevotella* increased, while RA of *Solobacterium*, *Bacillus*, and *Corynebacterium* decreased. These microbiota alterations may be directly linked to the incidence of diarrhea.

In this review, all data on jejunal mucosa-associated microbiota were obtained under consistent experimental conditions by the same research group. Therefore, potential variability due to pig genetic background, F18^+^ *E. coli* strain, experimental setup, sampling procedure, or microbiome sequencing method is minimized. Consistent changes in microbial composition at the phylum level were observed across studies^[36,37]^, with similar patterns also found at the family^[23,36,37]^, genus^[23,30,36]^, and species levels^[36,37]^. Nonetheless, some inconsistencies were noted, likely reflecting the microbiota’s sensitivity to factors such as immune status and diet composition. For example, *Bacteroidetes* RA increased in response to F18^+^ *E. coli* in the study by Garavito-Duarte *et al.*^[27]^, but decreased in the study by Duarte and Kim^[23]^. Similar inconsistencies were seen with Prevotellaceae^[27,36]^ and *Selenomonas*^[28,30,36]^, which may stem from differences in feed composition or individual pig variation. These findings highlight the complexity of the jejunal mucosa-associated microbiota and underscore the need for further research to resolve these inconsistencies regarding the impact of F18^+^ *E. coli* challenge on intestinal microbiota.

## IMPACTS OF F18^+^ *E. coli* CHALLENGE ON IMMUNITY AND OXIDATIVE STRESS IN THE JEJUNAL MUCOSA

Data on immune response and oxidative stress in the jejunal mucosa were collected from 10 studies involving F18^+^ *E. coli* challenges^[9,19,23,27-31,36,37]^. These data reflect host immune responses, including inflammatory reactions, triggered by F18^+^ *E. coli* infection. On average, these studies reported increases of 14.9%, 10.9%, 9.2%, and 19.7% in TNF-α, IL-8, immunoglobulin A (IgA), and immunoglobulin G (IgG) levels, respectively, in the jejunal mucosa of pigs [Table 4]. The upregulation of immune responses is likely due to the increased RA of Gram-negative bacteria^[23,28,30]^, which have LPS in their outer membranes^[45]^. LPS binds to a receptor complex (comprising TLR4, MD2, and CD14) on porcine epithelial cells^[93,94]^. This complex activates the MyD88-dependent pathway, which in turn stimulates NF-κB activation^[55]^. NF-κB regulates the expression of pro-inflammatory genes and mediates the synthesis of TNF-α, IL-6, and IL-8^[55]^. The F18^+^ *E. coli* antigen is recognized by dendritic cells in Peyer’s patches and mesenteric lymph nodes, where the antigen is presented to helper T cells^[95,96]^. These helper T cells subsequently activate naïve B cells, inducing the secretion of IgA and IgG. Moreover, enterotoxins STa and LT secreted by F18^+^ *E. coli* stimulate the production of IL-6 and IL-8 via the cAMP-mediated pathway^[97,98]^. The STb enterotoxin indirectly promotes the secretion of TNF-α, IL-6, and IL-8 by activating NF-κB through Ca^2+^ influx^[99]^.

**Table 4:** Changes in jejunal mucosal immunity and oxidative stress in nursery pigs challenged with F18^+^ *E. coli*

**Ref.**		**Inoculation BW (kg)**		**Inoculation age (d)**		**Days post inoculation (d)**	**Change^1^ (Δ%)**						
							**TNF**-**α**	**IL-6**	**IL-8**	**IgA**	**IgG**	**MDA**	**Protein carbonyl**
Duarte and Kim^[23]^		NA		28		21	13.9	-	-	-	-	-	36.1^*^
Garavito-Duarte *et al.*^[27]^		6.3		28		21	–22.3	–9.1	–2.1	–6.6	–12.1	–2.4	27.6
Gormley *et al.*^[28]^		6.4		28		21	–8.9	–43.2	11.5	2.9	5.4	35.3	24.2
Deng *et al.*^[31]^		6.6		28		18	–8.2	–18.9	–27.3^*^	–11.2	-	17.0	2.9
Duarte *et al.*^[9]^		6.9		28		21	26.7	-	-	-	-	-	67.9^*^
Xu *et al.*^[30]^		7.1		28		21	12.5	–6.7	83.3	42.4	24.7	–1.4	47.4^*^
Jang *et al.*^[29]^		7.2		28		21	42.7	-	–2.8	18.5	60.7	41.9	66.5
Duarte *et al.*^[36]^		8.2		28		13	10.4	45.3^*^	3.0	-	-	215.4^*^	7.6
Duarte *et al.*^[37]^		8.2		NA		21	17.1	-	10.8	-	-	87.4^*^	10.4
Sun *et al.*^[19]^		8.3		34		12	64.8^*^	-	-	-	-	12.1	-
**Number of studies^2^**							**Average change^3^ (Δ%)**						
**TNF-α**	**IL-6**	**IL-8**	**IgA**	**IgG**	**MDA**	**Protein carbonyl**	**TNF-α**	**IL-6**	**IL-8**	**IgA**	**IgG**	**MDA**	**Protein carbonyl**
10	5	7	5	4	8	9	14.9	–6.5	10.9	9.2	19.7	50.7	32.3

^1^Percentage changes in immune and oxidative responses were calculated by comparing the positive control (*E. coli*-challenged group) with the negative control (non-challenged group). ^2^Number of studies used to calculate the average change in each indicator. ^3^The average change was calculated as the arithmetic mean. ^*^P < 0.05. *E. coli*: *Escherichia coli*; BW: body weight; TNF-α: tumor necrosis factor-alpha; IL-6: interleukin-6; IL-8: interleukin-8; IgA: immunoglobulin A; IgG: immunoglobulin G; MDA: malondialdehyde; NA: not available.

These immune responses act as defense mechanisms against F18^+^ *E. coli* infection but also negatively impact intestinal health. For instance, TNF-α and IL-8, secreted by immune cells, disrupt intestinal barrier function by damaging tight junction proteins such as occludin and claudin^[57-59]^. TNF-α also induces apoptosis in both intestinal epithelial cells and pathogens, contributing to reduced villus height (VH) and microbial dysbiosis^[19]^. Similarly, IL-8 promotes oxidative stress by generating reactive oxygen species (ROS), which damage intestinal cells^[59]^. Elevated levels of IgA and IgG have also been associated with dysbiosis and intestinal barrier disruption^[100,101]^.

Regarding IL-6, the F18^+^ *E. coli* challenge does not consistently increase IL-6 levels in the jejunal mucosa of pigs. Among the relevant studies^[27,28,30,31,36]^, the study by Duarte *et al.* reported an increase in IL-6^[36]^. This inconsistency may be attributed to differences in the timing of post-inoculation sampling (13 *vs.* 18 or 21 days). IL-6 typically rises and falls earlier than IL-8 in response to infection^[102]^. Although both cytokines are regulated via similar pathways involving NF-κB, IL-8 gene expression involves more complex regulatory mechanisms than IL-6^[103]^.

Across the studies using F18^+^ *E. coli* challenge models, malondialdehyde (MDA) and protein carbonyl levels in the jejunal mucosa increased by an average of 50.7% and 32.3%, respectively^[9,19,23,27-31,36,37]^. These elevated levels serve as markers of both host defense and oxidative damage. ROS generated during the immune response oxidize lipids and amino acids in both host and bacterial cells^[104]^. F18^+^ *E. coli*, along with inflammatory cytokines, activates immune cells such as neutrophils and macrophages, which produce ROS^[105]^. These ROS eliminate pathogens by oxidizing their cellular components, generating MDA and protein carbonyl as by-products^[106]^. However, ROS also oxidize host cell components, leading to structural damage, reduced VH, and compromised intestinal integrity^[107]^.

## IMPACTS OF F18^+^ *E. coli* CHALLENGE ON JEJUNAL MORPHOLOGY

Jejunal morphology data from 14 studies involving F18^+^ *E. coli* challenge were reviewed^[9,23,27-30,36,37]^. On average, F18^+^ *E. coli* challenge resulted in a 10.2% and 10.7% decrease in VH and VH-to-crypt depth (CD) ratio (VH:CD), respectively, and a 0.9% and 35.4% increase in CD and crypt cell proliferation in the jejunum [Table 5]. These parameters serve as indicators of morphological development and the extent of damage caused by F18^+^ *E. coli* infection. F18^+^ *E. coli* reduces VH in the jejunum primarily through inflammation- and oxidative stress-induced apoptosis, which in turn impairs nutrient absorption^[9]^. Although inflammation and oxidative stress could theoretically reduce CD as well^[19]^, the reviewed studies show inconsistent effects of F18^+^ *E. coli* on CD. This inconsistency may be explained by structural differences between villi and crypts: villi are finger-like projections exposed to both luminal digesta and the mucosa surface, while crypts are located at the base of the villi and are less exposed to the luminal environment. These characteristics may make crypts less susceptible to F18^+^ *E. coli*-induced damage. The observed reduction in the VH:CD ratio is mainly driven by the greater reduction in VH compared to the increase in CD. Ki-67^+^ cells, which are present in all active cell cycle phases except G0, serve as a marker of cell proliferation. The increased proportion of Ki-67^+^ proliferative cells in response to F18^+^ *E. coli* suggests tissue damage by the pathogen and a corresponding activation of repair mechanisms^[108]^. The morphological changes induced by F18^+^ *E. coli* ultimately impair nutrient absorption and increase nutrient loss due to the energy demands of tissue repair in nursery pigs.

**Table 5:** Changes in jejunal morphology in nursery pigs challenged with F18^+^ *E. coli*

**Ref.**	**Inoculation BW (kg)**	**Inoculation age (d)**	**Days post inoculation (d)**	**Change^1^ (Δ%)**			
				**VH**	**CD**	**VH:CD**	**Ki-67^+^**
Duarte and Kim^[23]^	NA	28	21	–12.1^*^	7.2	–17.7^*^	7.8
Garavito-Duarte *et al.*^[27]^	6.3	28	21	–13.2^*^	–7.3	–6.1	25.0^*^
Gormley *et al.*^[28]^	6.4	28	21	–11.5^*^	8.1	–18.5^*^	3.8
Deng *et al.*^[31]^	6.6	28	18	–3.8	–9.7^*^	14.3^*^	2.3
Duarte *et al.*^[9]^	6.9	28	21	–25.2^*^	3.3	–28.8^*^	–9.0
Xu *et al.*^[30]^	7.1	28	21	–16.3	8.0	–22.4^*^	30.5^*^
Liu *et al.*^[26]^	7.1	25	5	–5.0^*^	4.9	–16.3^*^	-
			11	–4.9	11.3^*^	–14.6^*^	-
He *et al.*^[25]^	7.1	28	7	–2.6	2.6	0.9	-
Jang *et al.*^[29]^	7.2	28	21	–9.9	7.5	–15.0^*^	44.0^*^
Wong *et al.*^[34]^	7.4	28	5	–14.5	–2.5	–12.4	-
			21	–12.1	–6.7	–5.9	-
Kim *et al.*^[32]^	7.9	NA	5	–19.1^*^	–8.9	–11.5	-
			11	–8.9	–6.3	–5.3	-
Duarte *et al.*^[36]^	8.2	28	13	–11.8^*^	11.5^*^	–20.3	16.5^*^
Duarte *et al.*^[37]^	8.2	NA	21	–2.2	0	–2.3	24.4^*^
Sun *et al.*^[19]^	8.3	34	12	–8.2^*^	–7.9^*^	–0.6	-
**Number of studies^2^**				**Average change^3^ (Δ%)**			
**VH**	**CD**	**VH:CD**	**Ki-67^+^**	**VH**	**CD**	**VH:CD**	**Ki-67^+^**
17	17	17	9	–10.2	0.9	–10.7	35.4

^1^Percentage changes in jejunal morphology were calculated by comparing the positive control (*E. coli* challenged group) to the negative control (non-challenged group). ^2^The number of studies included in the average calculation for each morphological parameter. ^3^Averages were calculated as arithmetic means. ^*^P < 0.05. *E. coli*: *Escherichia coli*; BW: body weight; VH: villus height; CD: crypt depth; VH:CD: villus height-to-crypt depth ratio; Ki-67^+^: proportion of Ki-67^+^ proliferative cells in the crypt, indicating cell proliferation; NA: not available.

## IMPACTS OF F18^+^ *E. coli* CHALLENGE ON OTHER PARTS OF THE INTESTINE IN PIGS

Although the jejunum is the primary site affected by F18^+^ *E. coli*, the negative impacts of this infection on the ileum - particularly those involving microbial alterations and inflammation - have also been investigated, due to the presence of F18 receptors on the ileal epithelium. In a study by Li *et al.*, the RA of *Escherichia* and Enterobacteriaceae, which include pathogenic species, increased in response to F18^+^ *E. coli* challenge^[33]^. Conversely, the RA of *Lactobacillus*, a genus comprising beneficial bacteria, was reduced in the ileum of infected pigs. This microbial imbalance, or dysbiosis, may promote the secretion of pro-inflammatory cytokines. The F18^+^ *E. coli* challenge also elevated IL-8 levels and secretory IgA levels in the ileal mucosa of nursery pigs^[18]^. At the gene expression level, the relative mRNA levels of IL-6 and TNF-α in the ileal mucosa were also upregulated following F18^+^ *E. coli* challenge^[33,109]^. Inflammation induced by the infection damaged ileal morphology and downregulated the expression of tight junction protein genes, potentially compromising intestinal barrier integrity^[18,109]^.

Research focusing on the large intestine remains limited, as F18^+^ *E. coli* primarily targets the small intestine due to the distribution of the F18 receptor. However, Duarte *et al.* reported a reduction in the RA of *Prevotella stercorea* in feces following F18^+^ *E. coli* challenge^[9]^. This species is considered beneficial due to its role in fermenting dietary fiber and producing SCFAs. Similarly, the RA of *Selenomonas lipolytica*, another SCFA-producing, fiber-fermenting bacterium, was also found to decrease^[23]^. These reductions in beneficial bacterial populations mirror those observed in the small intestine, although the specific genera affected differ. These discrepancies may stem from inherent differences in the microbial community composition and substrate availability between the small and large intestines^[80]^.

## CONCLUSION

This review outlines the mechanisms and consequences of F18^+^ *E. coli* challenge on intestinal health in the jejunal tissue and mucosa of nursery pigs. F18^+^ *E. coli* interacts with the jejunal epithelium through its structural components. The F18 fimbriae, unique to this strain, facilitate attachment and colonization within the pig intestine. After colonization, the bacterium secretes enterotoxins, inducing diarrhea via electrolyte imbalance and ultimately reducing pig productivity. According to the reviewed literature, F18^+^ *E. coli* challenge increases diarrhea incidence by 28.3% and fecal score by 0.7 points (on a 1 to 5 scale), leading to reductions in ADG (24.1%), ADFI (12.5%), and G:F (14.9%).

These productivity losses are not solely attributable to diarrhea, but also to the broader negative impacts on intestinal health. F18^+^ *E. coli* alters the composition of the jejunal mucosa-associated microbiota by reducing populations of beneficial bacteria and increasing harmful ones. This shift triggers an immune response in the intestinal epithelium, leading to inflammation and oxidative stress. These responses damage epithelial cells and induce morphological changes. Specifically, F18^+^ *E. coli* challenge has been associated with decreased RA of beneficial genera such as *Lactobacillus* and *Bifidobacterium*, and increased RA of harmful genera including *Helicobacter* and *Prevotella*. The challenge also elevated levels of TNF-α (14.9%), IL-8 (10.9%), IgA (9.2%), IgG (19.7%), MDA (50.7%), and protein carbonyl (32.3%). These immune and oxidative responses compromise jejunal morphology, evidenced by reductions in VH (10.2%) and VH:CD (10.7%) and an increase in Ki-67^+^ proliferative cells (35.4%). Collectively, these changes contribute to impaired growth performance in nursery pigs.

## DECLARATIONS

### Copyright

© The Author(s) 2025.
